# Insight Into an Outbreak of Canine Distemper Virus Infection in Masked Palm Civets in China

**DOI:** 10.3389/fvets.2021.728238

**Published:** 2021-11-03

**Authors:** Ning Shi, Le Zhang, Xiuhua Yu, Xiangyu Zhu, Shu Zhang, Daining Zhang, Ming Duan

**Affiliations:** ^1^Key Laboratory of Zoonosis Research, Ministry of Education, Institute of Zoonosis, College of Veterinary Medicine, Jilin University, Changchun, China; ^2^Changchun Veterinary Research Institute, Chinese Academy of Agricultural Sciences, Changchun, China; ^3^Department of Pediatrics, The First Hospital of Jilin University, Changchun, China

**Keywords:** canine distemper virus (CDV), civet (*Viverridae*), China, phylogenetic analysis, zoonotic potential

## Abstract

In August 2019, a suspected outbreak of canine distemper was observed in a masked palm civet farm that also received stray civets and rescued wild civets in Henan Province of China. A virulent canine distemper virus (CDV) strain, named HN19, from vaccinated masked palm civets was the etiologic agent identified in this outbreak using RT-PCR and sequencing of the complete genome. Serological analysis indicated a lower positive rate of CDV-neutralizing antibody in wild civets than in captive civets. Phylogenetic analysis of viral hemagglutinin (H) and the complete genome showed high identities with Rockborn-like strains at the nucleotide (98.7~99.72%) and the closest nucleotide similarity with a strain that killed lesser pandas in China in 1997, but low identities with America-1 strains (vaccine strains). Most importantly, one distinct amino acid exchange in the H protein at position 540 Asp → Gly (D540G), which confers CDV with an improved ability to adapt and utilize the human receptor, was observed in HN19. This study represents the first reported outbreak of a Rockborn-like CDV strain infection in masked palm civets in China. Based on this report, the existence of Rockborn-like strains in Chinese wild animals may not only cause immune failure in captive animals, but may also confer increased zoonotic potential.

## Introduction

Morbilliviruses belong to the order *Mononegavirales*, the family *Paramyxoviridae* and include a group of highly pathogenic viruses, such as measles virus (MeV), rinderpest virus (RPV) and canine distemper virus (CDV). In contrast to host-specific Mev and RPV, CDV has higher genetic diversity and causes a highly contagious disease in a wide broad of animals, including dog, civet, phocine, ferret, lion, raccoon, fox, etc. (1–6). Thus, spillover and spillback transmission of CDV between domesticated animals and wildlife reservoir hosts has been documented (6). Even more alarmingly, the host range of CDV has been expanded to other species that are evolutionarily more distant to canids, such as Asian marmots (*Marmota caudata*) and Japanese monkeys (*Macaca fuscat*) (7, 8).

The hemagglutinin (H) gene of CDV encodes the receptor-binding protein. Currently, the signaling lymphocyte activation molecule (SLAM) (also known as CD150) and the cell adhesion molecule Nectin-4 (also known as poliovirus receptor-like protein 4, PVRL4) are known to engage with H protein (9, 10). Of these, SLAM, as the principal cellular receptor for morbilliviruses, has been shown to be critical for host susceptibility and virus entry, whereas nectin-4 is required for clinical disease and efficient virus shedding (11, 12). A recent study revealed that a substitution in CDV H protein at residue 540, Asp to Gly (D540G), is sufficient to allow CDV to bind to human SLAM *in vitro*, which could cause CDV to potentially adapt human target cells (13).

The masked palm civet *Paguma larvata* (order Carnivora, family *Viverridae*) is distributed in tropical and subtropical Asia (14). In China, masked palm civets are raised as new farm animals mainly in the southern provinces for meat production. Civets have been thought to be potential intermediate hosts that can provide an effective way for the virus, such as severe acute respiratory syndrome (SARS) which appeared in southern China in 2003, to spread from animals to humans (15). In addition, several studies have shown that the masked palm civet may potentially be involved in transmission of some zoonotic pathogens such as *Salmonella enterica, Bartonella henselae*, and *Toxoplasma gondii* (16–18).

Up to now, in China, there are none reports of Rockborn-like strain detected from a civet. The present study aimed to investigate the CDV infection in civets, analyze the genotypes of epidemic strains, and report the cross-species transmission of CDV.

## Materials and Methods

### Sample Collection

In August 2019, sudden and unexplained fever, severe lethargy and weakness, loss of appetite, mild-to-marked upper respiratory disease and neurological dysfunction (severe lethargy, loss of appetite, epilepsy and twitching) were observed in a masked palm civet farm that also received stray civets and rescued wild civets in Luoyang city, Henan Province. Seventy percent of the sick civets died on days 7~10 of the illness after accepting new wild animals. Although the immune status of the wild and stray animals was unclear, all the cultured animals had been vaccinated with the American-1 strain. We randomly collected ocular, nasal and rectal swab specimens, and serum samples from 44 healthy and 57 sick civets. Fresh tissues harvested from 9 dead animals were used for histopathological analysis, immunohistochemical analysis and viral isolation. After collection, the samples were immediately transported to our laboratory in an icebox for further use.

### Viral Detection and Isolation

All samples were stored at −80°C and tested, initially by Anigen Rapid CDV Ag Test Kit (BioNote, Inc.Gyeonggi-do, South Korea) according to the manufacturer's instructions. Total RNA was extracted from the tissues with a QIAamp Viral RNA Mini Kit (Qiagen, USA). The RNA was converted into cDNA using a Vazyme HiScript II 1st Strand cDNA Synthesis Kit (Vazyme Biotech Co., Ltd., China) in accordance with the manufacturer's instructions. To validate the presence of CDV, reverse transcription polymerase chain reaction (RT-PCR)/PCR was performed to detect a 681 bp RT-PCR amplicon encompassing the fusion protein signal-peptide (Fsp)-coding region (405 bp) using a previously described primer (19). For virus isolation, the tissues were homogenized in 20 % (w/v) sterile phosphate-buffered saline (PBS, pH 7.4) and filtered through 0.22 um membrane filters. Supernatants of homogenized lung filtered through 0.22 um membrane filters were used to inoculate Vero-raccoon dog-SLAM cells as described previously (20), and the cytopathogenic effect was observed within 120 hours. Vero-raccoon dog-SLAM cells were constructed by the authors and were maintained in DMEM containing 5% FBS (data not published).

### Electron Microscopic Analysis

The Vero-SLAM cells at 4 days post-infection were used for electron microscopic analysis. Cell supernatants were centrifuged at 12,000 × g for 5 min at 4°C. Virus-containing supernatants were negatively stained and examined using transmission electron microscopy (TEM) (21).

### Whole Genome Sequencing and Phylogenetic Analysis

The complete H gene of the CDV isolate and virus contained in brain and lung samples from deceased civets was amplified for sequencing by RT-PCR using H gene specific primers (20). The entire genome of civets CDV isolated with Vero-raccoon dog-SLAM cells was amplified and sequenced using a set of 15 primer pairs to generate overlapping PCR amplicons (20). Multiple sequence alignments were performed and sequence similarities determined using DNASTAR software. The neighbor-joining (NJ) method with 1,000 bootstrap replicates was used to construct a phylogenetic tree in MEGA version 7.0 (22).

### Histopathological Examination and Immunohistochemical Analysis

For histological studies, samples intended for histopathological examination (brain, lung, heart, liver, kidney and testicle) were fixed in 4% buffered formaldehyde solution and 4 μm thick paraffin sections were mounted on silane-coated slides. Slides were stained with hematoxylin and eosin (HE) according to the standard histopathological procedure.

Immunohistochemical analysis was performed in the same tissues using the chain polymer-conjugated method (2). Briefly, slides were deparaffinised and rehydrated. After antigen retrieval and endogenous peroxidase blocking, slides were incubated with an anti-CDV nucleoprotein monoclonal antibody at a 1:100 dilution (VMRD Inc., Pullman, WA. Cat. P180221). Then, the slides were incubated using the Dako REAL EnVision Detection System (Dako, Glostrup, Denmark), at 37°C as a secondary antibody, and the positive antigen-antibody complex was then visualized by labeling with 3, 3′-diaminobenzidine tetrahydrochloride (DAB) and counterstaining with Mayer's hematoxylin. Positive control slide was performed on lung tissue from a CDV-infected dog that was positive in the RT-PCR assay, while the Tris-HCl buffer instead of the primary antibody was used as a negative control.

### Virus Neutralizing Antibody Titers (VNT)

CDV virus neutralizing (VN) antibody titers were determined in Vero-SLAM cells using a TCID50 microtiter assay as previously described (23) with minor modifications. Briefly, serial 2-fold dilutions of heat-inactivated serum starting at 1:8 were added to ~100 TCID_50_ of vaccine strain CDV3 (EU726268) and tested in quadruplicate. The titers were calculated using the method of Reed and Muench.

## Results

### Fatal CDV Infection in Masked Palm Civets

RT-PCR analysis was used to detect viral nucleic acid. Tissue samples from all of the dead civets were all CDV infection-positive. The swab samples (*n* = 101) showed a detection rate of 56.44%. Serological analysis indicated a lower positive rate of CDV-neutralizing antibody in wild civets than in captive civets, regardless of the health condition. The results of all samples tested by PCR and neutralizing antibodies are summarized in Table 1. The cytopathogenic effect (CPE) was observed in Vero-SLAM cells within 96~120 h after infection from positive lung tissue. Numerous spherical, enveloped virus particles of ~200 nm in diameter were observed by negative-staining electron microscopy in isolated strain (named HN19) (data not shown).

**Table 1:** Detection of civet CDV in sick and healthy animals using PCR and SN test.

**Species**	**Total No**.	**Vaccination status**	**Health status(dead/total)**	**Rate**	
				**PCR^+^ (swabs)**	**SN^+^ (blood)**
Wild and stray	30	Unknown	Healthy (0/4)	1/4	2/4
			Sick (26/26)	25/26	3/26
Captive	291	Vaccinated	Healthy (0/260)	2/40	38/40
			Sick (14/31)	29/31	28/31
Total	321			57/101	71/101

+ *Positive*.

### Histopathological Analysis

**Figure 1:**
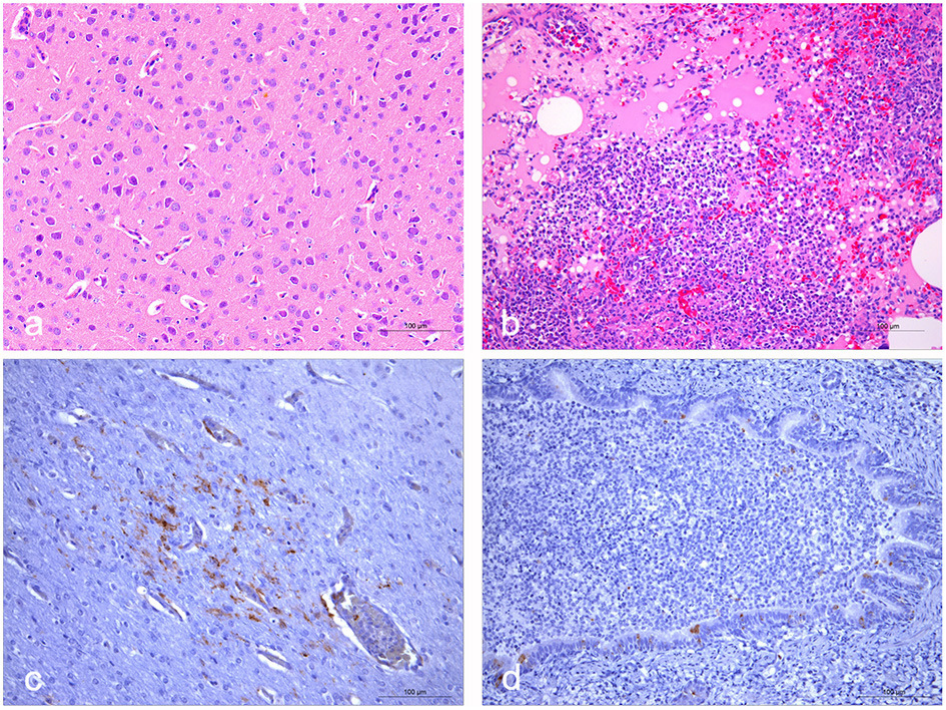
Histopathological and immunohistochemical staining of lungs and brains of fresh dead civets. **(a)** Microscopic examination of brain samples showed neuronal degeneration and necrosis, perivascular edema. **(b)** The alveolar wall capillaries were dilated and congested, and part of the alveolar cavity was filled with abundant inflammatory exudates containing edematous fluid and lymphocytes. Moreover, lymphocytes and macrophages can be seen in the bronchus. **(c)** Immunohistochemical staining showed that some nerve cells in the brain tissue were positive for CDV. **(d)** CDV was positively detected in some bronchial epithelial cells. The scale bar indicates 100 μm.

Briefly, histopathologic findings in the brain included neuronal degeneration and necrosis, perivascular edema (Figure 1a). Interstitial pneumonia with infiltrates of inflammatory cells comprising lymphocytes and macrophages can be seen in the bronchus (Figure 1b). Immunohistochemical analysis revealed the CDV antigen in some areas of the lung and brain (Figures 1c,d). Additionally, generalized more severe lymphocyte depletion was found in the spleen, liver, intestine, while no obvious pathological changes were found in kidney (data not shown).

### Phylogenetic Analysis

**Figure 2:**
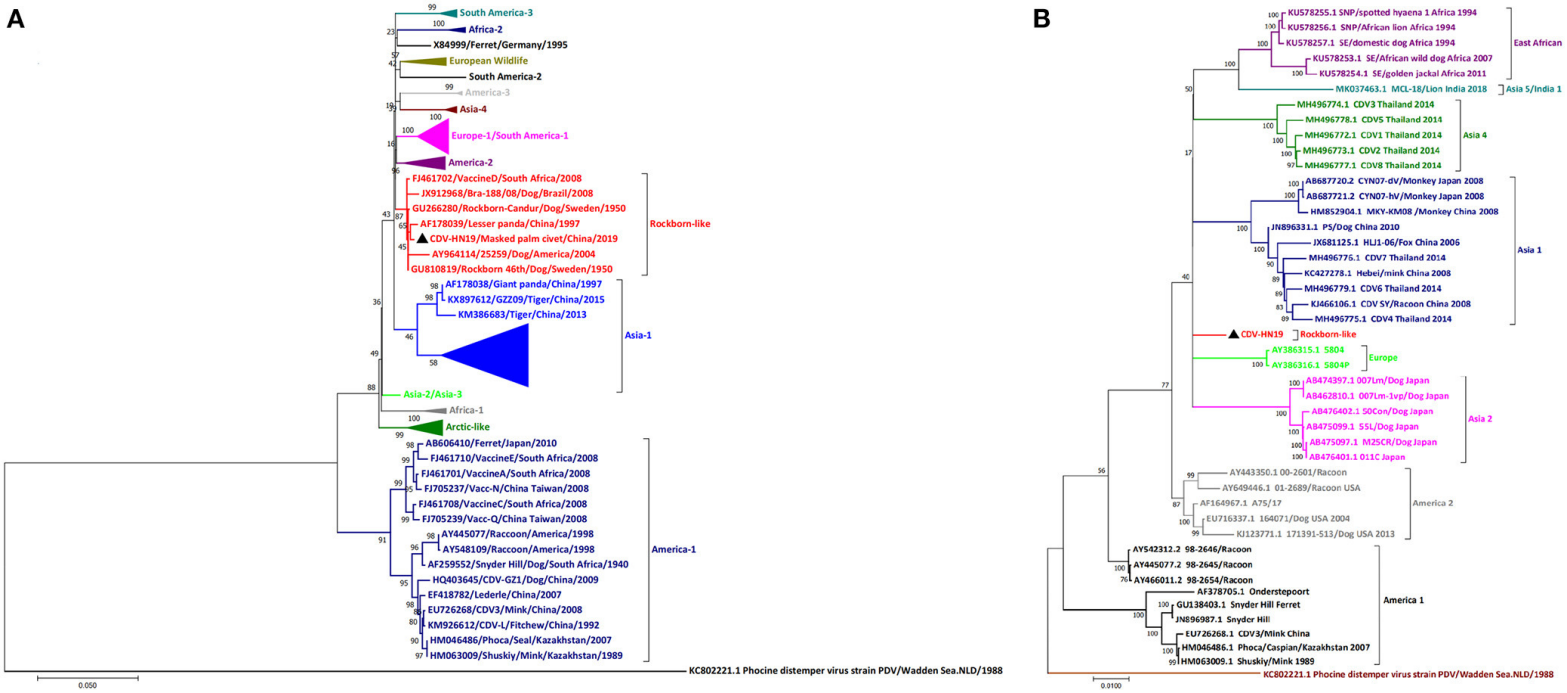
Phylogenetic analyses of CDV HN19 strain. **(A)** Phylogenetic analyses of the nucleotide sequences of the complete genome of CDV HN19 strain. **(B)** Phylogenetic analyses of H gene nucleotide sequences of CDV HN19. Evolutionary history was inferred using the maximum likelihood method with the Tamura-Nei model and gamma-distributed rate heterogeneity in MEGA 7. The percentage of replicates in which the associated virus clustered together in the bootstrap test (1,000 replicates) is shown next to the branch in each tree. The strain isolated in this study is identified by ▲. The percentage bootstrap support is indicated by the value at each node. Scale bar denotes nucleotide substitutions per site.

The complete viral genome of the HN19 strain was sequenced. This sequence has been deposited in GenBank under the accession number MT448054. Phylogenetic analysis and multiple sequence alignments based on the H gene sequence showed that HN19 strain belongs to the Rockborn-like strain cluster (Figure 2A). Sequence comparisons of the H gene of HN19 strain showed high identities with Rockborn-like strains at the nucleotide (98.7~99.72%) and the deduced amino acid (97.78~99.15%) levels (Table 2). Additionally, HN19 clustered with a strain that killed lesser pandas in China in 1997 (24) (Figure 2A). Sequence comparisons of the complete genome showed that HN19 had low sequence identities with America-1 strains (Figure 2B). The H gene sequence of the HN19 stain demonstrated a low nucleotide similarity (92.16~92.68%) with America-1 CDVs. Similarly, at the amino acid level of H gene, HN19 had slightly lower identity (89.48~90.60%) to America-1 CDVs (Table 2).

**Table 2:** Nucleotide and amino acid changes in the H gene of CDV isolated from the civet compared to published sequences.

**Lineages**	**Information**	**Strain**	**Position**									
			**Nucleotide**	**314**	**475**	**592–3**	**794**	**1,126–7**	**1,619**	**1,642–3**	**Nucleotide**	**Amino Acid**
			**Amino Acid**	**105**	**159**	**198**	**265**	**376**	**540**	**548**		
**Rockborn**	**Civet/China/2019**	**CDV-HN19**		**C(S)**	**C(L)**	**TT(F)**	**C(S)**	**AC(T)**	**G(G)**	**AT(M)**	**100%**	**100%**
	Dog/Sweden/1950	GU810819		T(L)			T(L)	AA(N)	**A(D)**	AC(T)	99.72%	99.15%
	VaccineD/South Africa/2008	FJ461702		T(L)			T(L)	AA(N)	**A(D)**	AC(T)	99.55%	98.64%
	Lesser panda/China/1997	AF178039						AA(N)	**A(D)**	AC(T)	99.49%	98.98%
	Dog/Brazil/2008	JX912968		T(L)			T(L)	AA(N)	**A(D)**	AC(T)	99.10%	98.47%
	Dog/America/2004	AY964114		T(L)			T(L)	AA(N)	**A(D)**	AC(T)	98.70%	97.78%
	Dog/Sweden/1950	GU266280		T(L)			T(L)	AA(N)	**A(D)**	AC(T)	99.66%	98.98%
Asia-1	Giant panda/China/1997	AF178038						AA(N)	**A(D)**	AC(T)	97.78%	97.60%
	Tiger/China/2015	KX897612						AA(N)	**A(D)**	AC(T)	97.67%	97.26%
	Tiger/China/2013	KM386683						AA(N)	**A(D)**	AC(T)	96.79%	96.03%
American-1				T(L)	A(I)	GT(V)	T(L)	GG(G)	**A(D)**	AC(T)	92.16%~92.68%	89.48%~90.60%
Others^*^				T(L)	&	+	T(L)	*	#	†	89.88%~97.83%	84.08%~96.38%

* *All remaining strains except Rockborn, Asia-1 and American-1*.

& *A(I), G(V)*.

+ *GT(V), AT(I), TC(S)*.

* *AT(I), AA(N), AG(S), AC(T), GG(G)*.

# *C(A), A(D)*.

† *AT(M), AC(T), CC(P)*.

The complete H gene sequence detected from brain and lung was as same as that detected from the isolate. Sequence analyses of H gene revealed that the same unique amino acid residues 105S and 265S existed in HN19, lesser panda isolates, giant panda isolates and two other tiger derivatives from China. Most importantly, the adaptive mutation D540G in the H protein was unique, which has been supposed to be required for adaptation of CDV to the human entry receptors (13). In addition, there were another two unique amino acid residues, 376T and 548M, which could be associated with vaccination failure and unusual clinical signs.

## Discussion

Previous reports have demonstrated that CDV is a lethal infectious agent to susceptible free-living and captive *Viverridae*, including civets (2, 25, 26). Here, we have documented a Rockborn-like CDV strain infection in masked palm civets in China. Because CDV had never appeared at this farm before, and this strain belonged to the Rockborn-like group along with another strain from wild animals, this outbreak might be attributed to stray animal contact or wild animal adoption. The Rockborn vaccine strain, as a canine isolate, was made on primary canine kidney cells in the 1950s (27). Compared with other CDV vaccines, Rockborn strain was considered to be less attenuated and less safe, and withdrawn from several markets after the mid-1990s (28). However, the isolation of Rockborn-like CDVs, respectively, from masked palm civets and lesser pandas in China suggests that Rockborn-like viruses are still circulating in the field or that vaccine-derived viruses were introduced in different carnivores on several occasions in China.

In this farm, vaccination failure was observed in 31/291 captive animals (10.65%) with an America-1 strain vaccination record. The efficacy of vaccines relies on the antigenic relatedness between the vaccine and the circulating field strains. The CDV H gene is the most heterogenic and antigenic variable among all different strains of CDV, which could result in the generation of antibodies with widely different neutralization capacity or vaccine breakdown. Phylogenetic analysis of the complete genome and viral H gene showed that HN19 shared low identities with America-1 strains, which could be a reason for this immune failure.

Morbilliviruses such as MeV and CDV use the species orthologs of CD150 and nectin-4 expressed on immune and epithelial cells, respectively, as receptors. Several studies have demonstrated that amino acid substitutions in the H protein may contribute to the expanded host range (13, 29). Among substitutions, 540 Asp → Gly (D540G) confers good fusion capacity to viral envelope proteins binding with human CD150, which may facilitate CDV adaptation to human target cells (13). The amino acid exchange D540G has been also observed in HN19 strain, a natural isolate from civets, implying that the zoonotic potential of CDV might be a matter of concern.

Through the investigation of CDV infection in civets in China, we formulated three hypotheses. The first was that a kind of Rockborn-like strain was circulating in wild animals in China. The second hypothesis was that the America-1 vaccine could not provide adequate protection for civets. Finally, the third hypothesis was Rockborn-like strain with the D540G mutation in Chinese wild animals might have zoonotic potential. Therefore, the information provided with this study emphasizes the need for the regular surveillance of wild animals, mandatory effective vaccination in wild animals and reduction of the increasing threat of CDV to animals and public health in China.

## Data Availability Statement

The datasets presented in this study can be found in online repositories. The names of the repository/repositories and accession number(s) can be found at: https://www.ncbi.nlm.nih.gov/genbank/, MT448054.

## Ethics Statement

The animal study was reviewed and approved by the farm owner and our research team made an agreement in a written way. The Ethical Review Committee approved this agreement. Furthermore, the study was approved by the farm owner and all experimental animals were conducted according to the International Guiding Principles for Biomedical Research. The protocol was approved by the Committee on the Ethics of Animal Experiments of the Jilin University. Written informed consent was obtained from the owners for the participation of their animals in this study.

## Author Contributions

NS, LZ, XY, XZ, SZ, DZ, and MD performed experimental study and analysis. NS and MD wrote the manuscript. All authors contributed to data collection, data analysis, manuscript revision, read, and approved the submitted version.

## Funding

This work was supported by the National Natural Sciences Foundation of China (No. 31970154).

## Conflict of Interest

The authors declare that the research was conducted in the absence of any commercial or financial relationships that could be construed as a potential conflict of interest.

## Publisher's Note

All claims expressed in this article are solely those of the authors and do not necessarily represent those of their affiliated organizations, or those of the publisher, the editors and the reviewers. Any product that may be evaluated in this article, or claim that may be made by its manufacturer, is not guaranteed or endorsed by the publisher.
